# Supplementary material for: Machine-learning-accelerated design of high-performance platinum intermetallic nanoparticle fuel cell catalysts
Source: Nat Commun. 2024 Jan 10;15:415. doi: 10.1038/s41467-023-44674-1 (PMC10776629; doi:10.1038/s41467-023-44674-1)
Supplement: Supplementary file 1 — Supplementary Information [file 41467_2023_44674_MOESM1_ESM.pdf]

Supplemental information

Machine-learning-accelerated design of high-performance platinum intermetallic nanoparticle fuel cell catalysts

Peng Yin[1,3], Xiangfu Niu[2,3], Shuo-Bin Li[1], Kai Chen[2], Xi Zhang[1], Ming Zuo[1], Liang Zhang[2]*, and Hai-Wei Liang[1]*

[1]Hefei National Research Center for Physical Sciences at the Microscale, Department of Chemistry, University of Science and Technology of China, Hefei 230026, China

[2]Center for Combustion Energy, School of Vehicle and Mobility, State Key Laboratory of Intelligent Green Vehicle and Mobility, Tsinghua University Beijing, 100084, China

[3]These authors contributed equally: Peng Yin, Xiangfu Niu

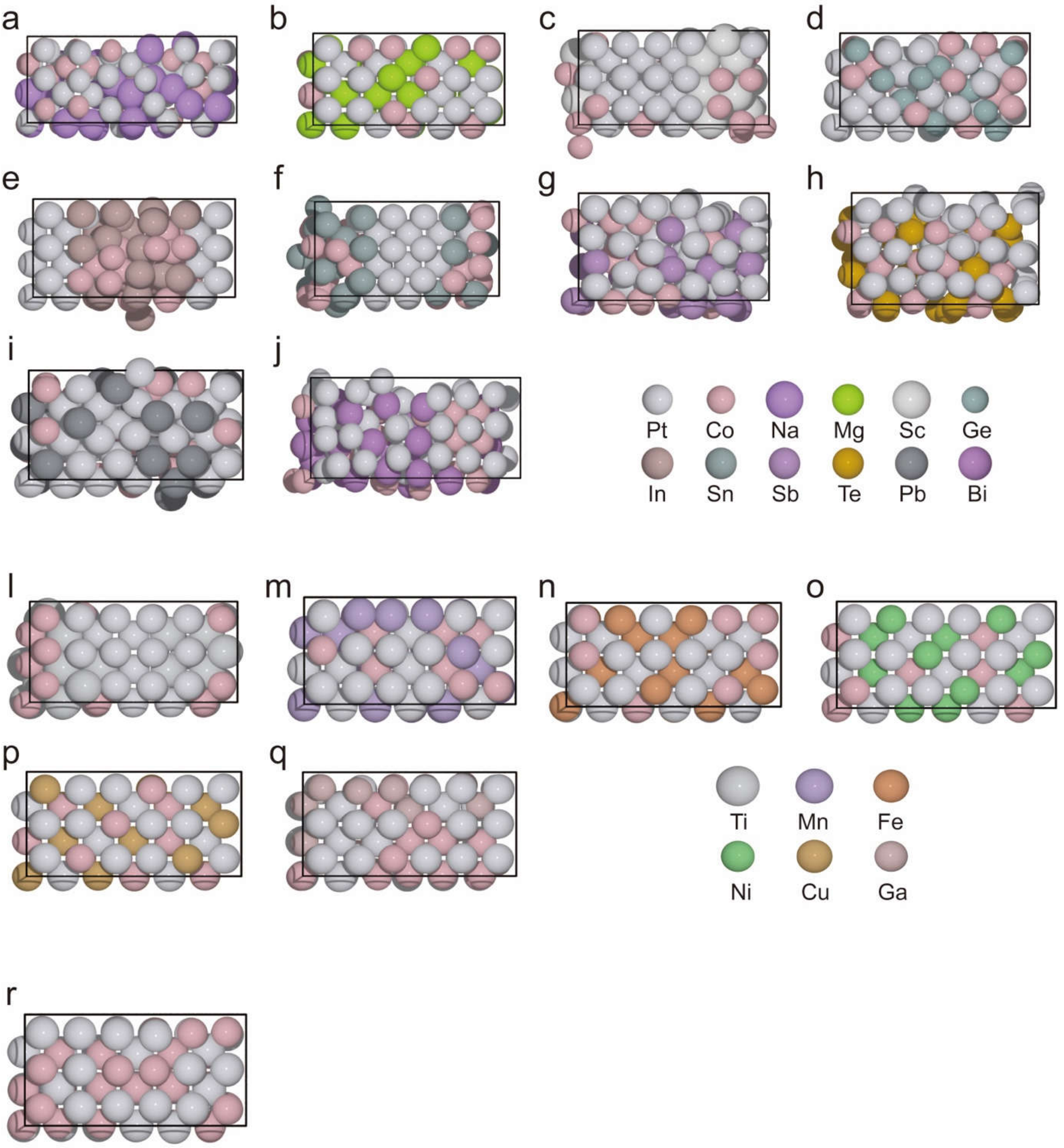

Supplementary Figure 1. All Pt_2CoM supercell models calculated by DFT in this work, including 10 insoluble alloy combiations Pt_2CoNa, Pt_2CoMg, Pt_2CoSc, Pt_2CoGe, Pt_2CoIn, Pt_2CoSn, Pt_2CoSb, Pt_2CoTe, Pt_2CoPb, Pt_2CoBi (**a-j**), 6 soluble alloy combiations Pt_2CoTi, Pt_2CoMn, Pt_2CoFe, Pt_2CoNi, Pt_2CoCu, Pt_2CoGa (**l-q**) and PtCo (**r**).

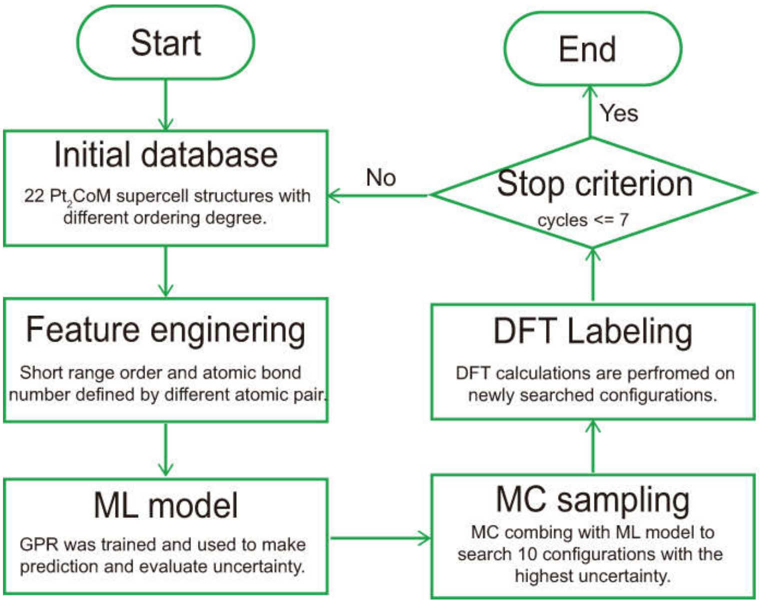

Supplementary Figure 2. Schematic workflow of the active learning to establish energy prediction ML model.

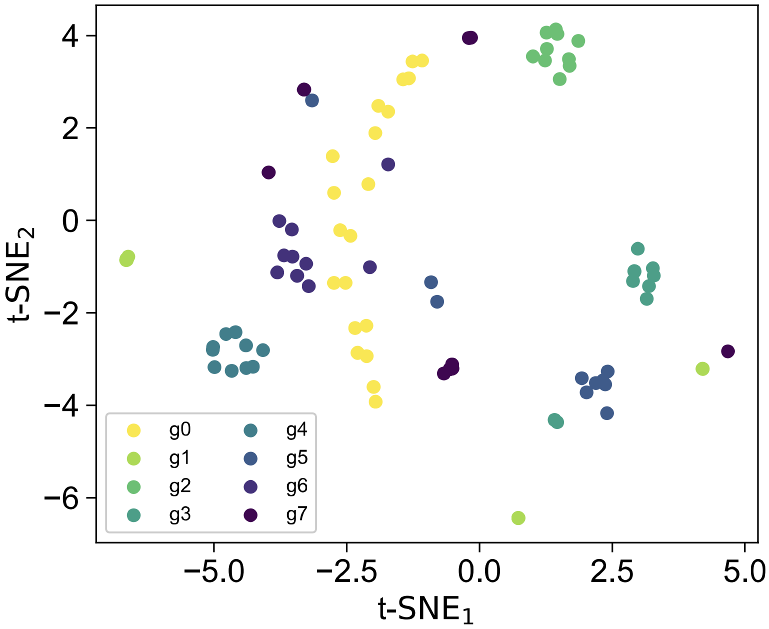

Supplementary Figure 3. All the DFT labeled Pt_2CoCu configurations in active learning process. Each datapoint represent one Pt_2CoCu configuration, x axis and y axis are two components of the ML feature downsized by t-SNE technology. Different colors indicate different cycles in active learning process from the initial dataset to the seventh cycles. In general, datapoints from the same cycle are clustered together, and datapoints from different cycles are separated, this distribution characteristic demonstrates the efficiency of active learning to avoid unbalanced sampling.

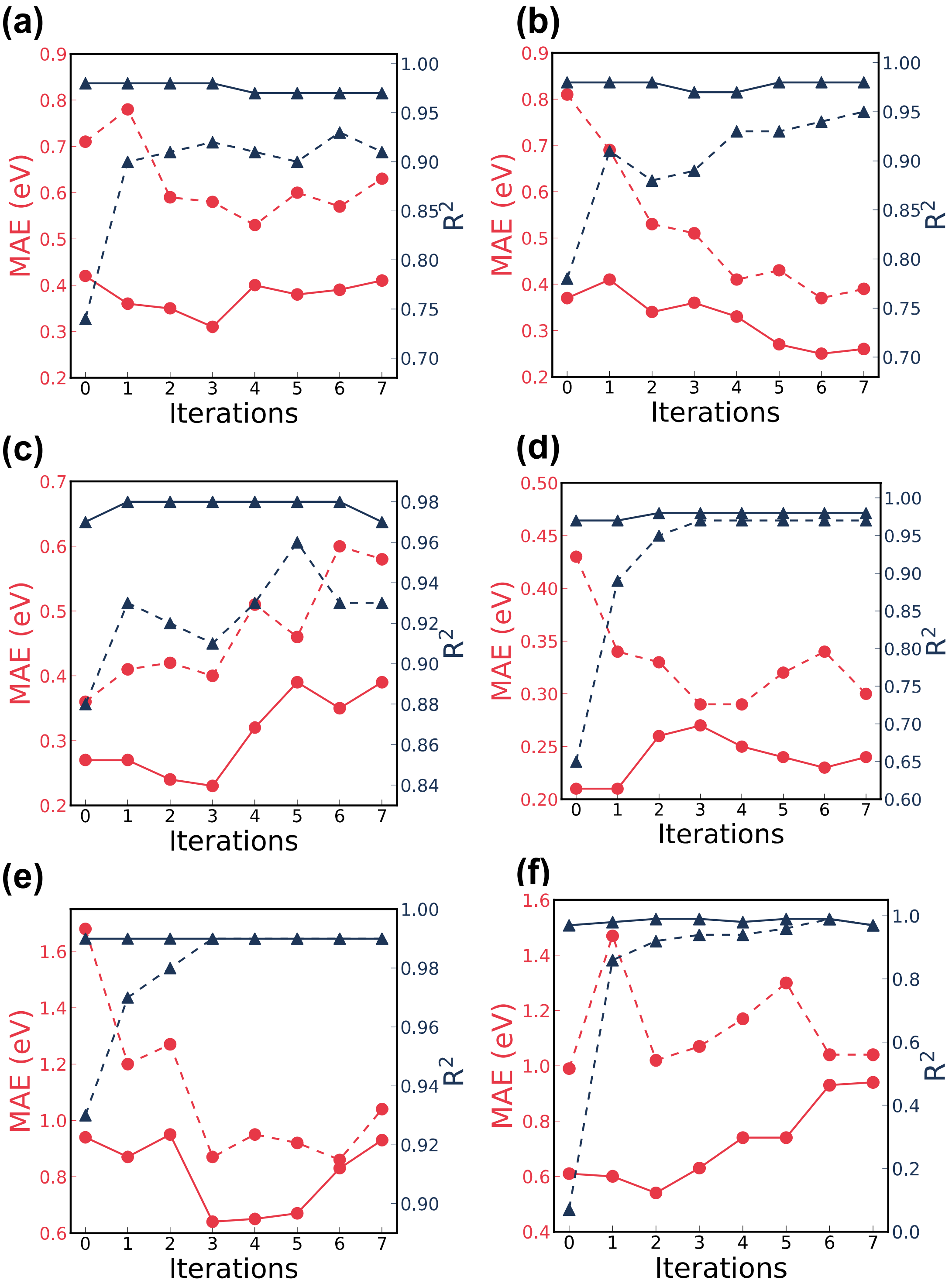

Supplementary Figure 4. Evolution of the mean absolute error (MAE) and coefficient of determination (R^2) of the machine learning model in the active learning process. The data was divided into two parts with 80% as the train set, and 20% as the test set. 10 random cross-validations were conducted and the mean values of MAE and R^2 of the ten times were used. The red line and red dotted line represent MAE of train set and test set; the blue line and blue dotted line represent R^2 of train set and test set. M is Cu **(a)**, Ni **(b)**, Mn **(c)**, Fe **(d)**, Ti **(e)**, and Ga **(f)**.

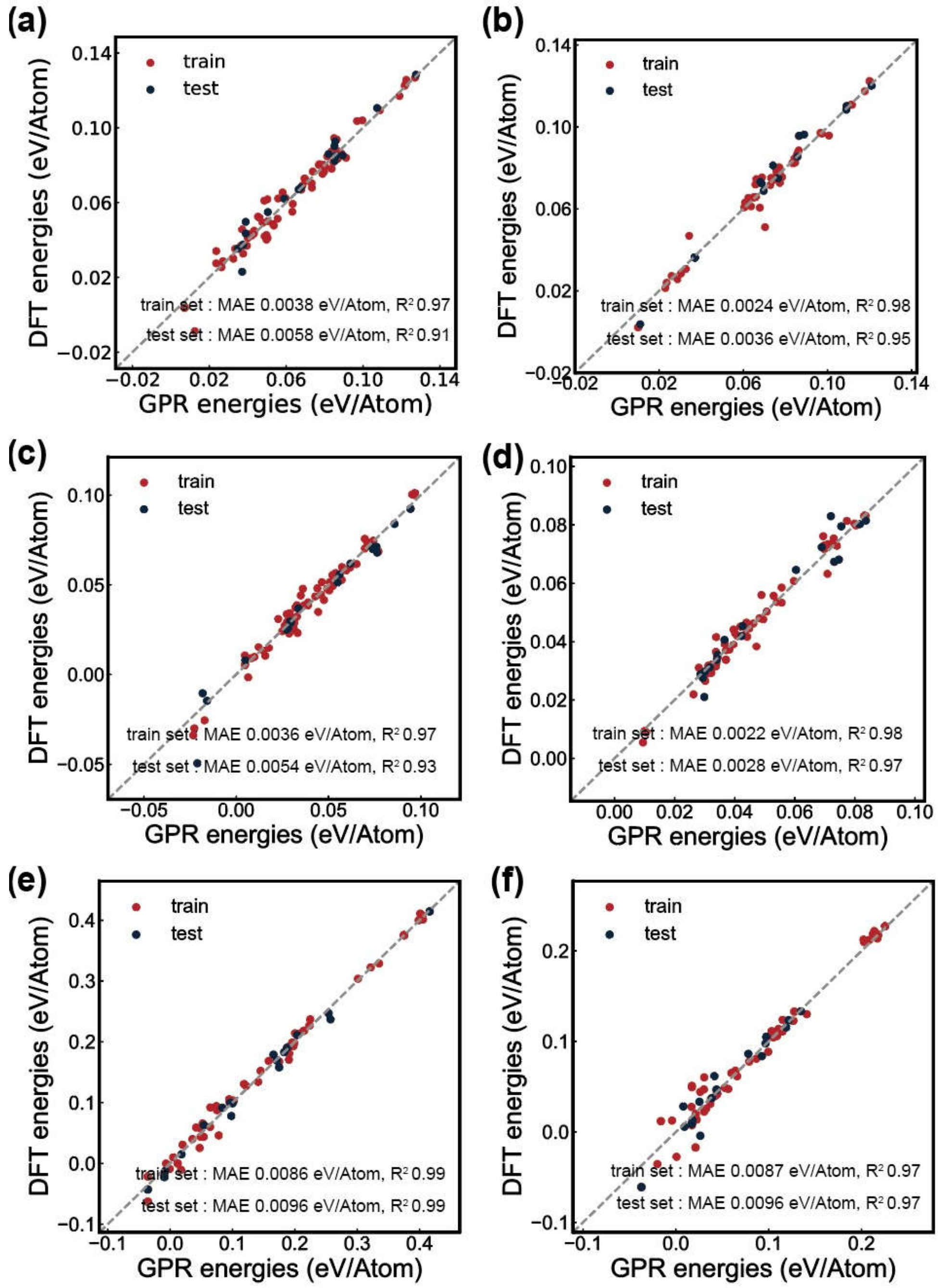

Supplementary Figure 5. Parity plot of six completed Pt_2CoM energy prediction model. M is Cu **(a)**, Ni **(b)**, Mn **(c)**, Fe **(d)**, Ti **(e)**, Ga **(f)**.

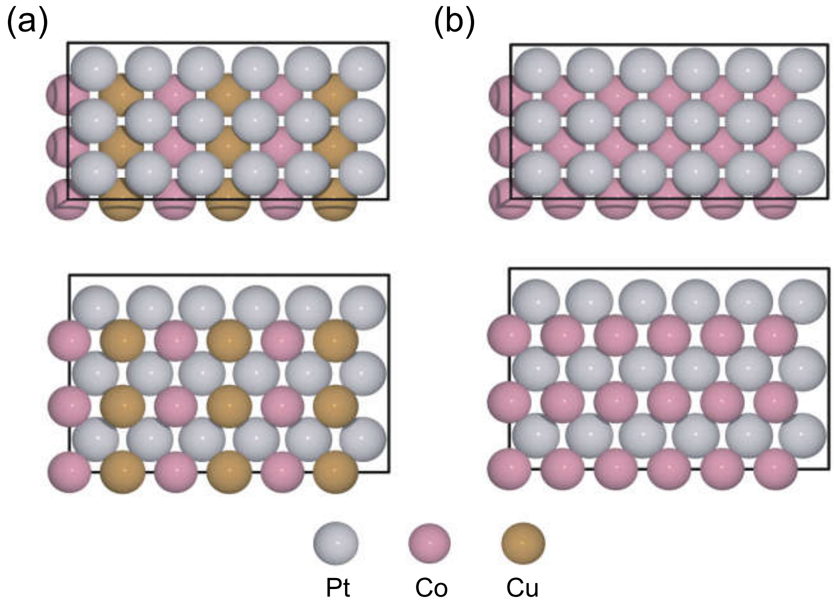

Supplementary Figure 6. Top and side views of ordered **(a)** Pt_2CoCu and **(b)** PtCo supercell models. For ordered Pt_2CoCu supercell model, there are 12 neighboring atoms around Pt atom in its first coordination shell, comprising 4 Pt atoms, 4 Co atoms, and 4 Cu atoms. There are also 12 neighboring atoms around a Co (Cu) atom in its first coordination shell, comprising 4 Pt atoms, 4 Cu(Co) atom and 4 Co(Cu) atoms. The probability of finding Pt atom around Co(Cu) atom in its first coordination shell is 8/12, the ratio of Pt is 1/2, which results in $\alpha^{\mathrm{Pt-(Co/Cu)}} = -1/3$. For ordered PtCo supercell model, the same analysis leads to a value of -1/3 for $\alpha^{\mathrm{Pt-Co}}$.

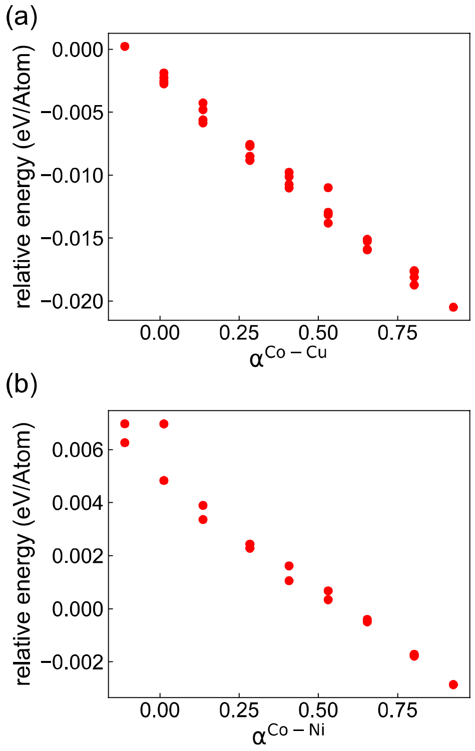

Supplementary Figure 7. Relative energies of Pt_2CoM as a function of α^{Co-M} at fixed $\alpha^{Pt-(Co/M)} = 1/3$. M is Cu **(a)** and Ni **(b)** respectively.

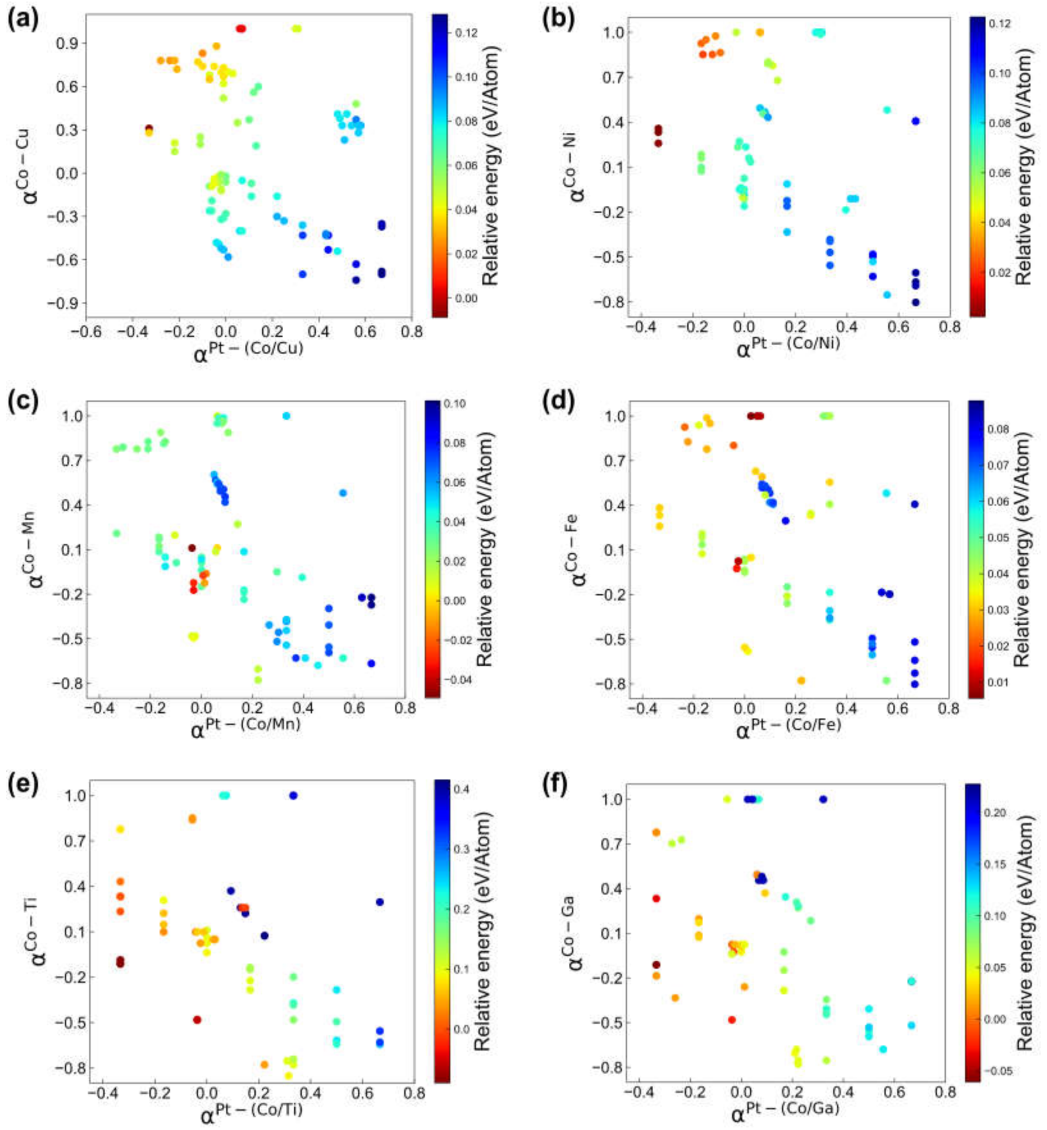

Supplementary Figure 8. Relationship between relative energies of Pt_2CoM as a function of two SRO parameters, x axis $\alpha^{Pt-(Co/M)}$ is SRO defined by Pt and Co/M, y axis α^{Co-M} is SRO defined by M and Co. M is Cu **(a)**, Ni **(b)**, Mn **(c)**, Fe **(d)**, Ti **(e),** and Ga **(f)**.

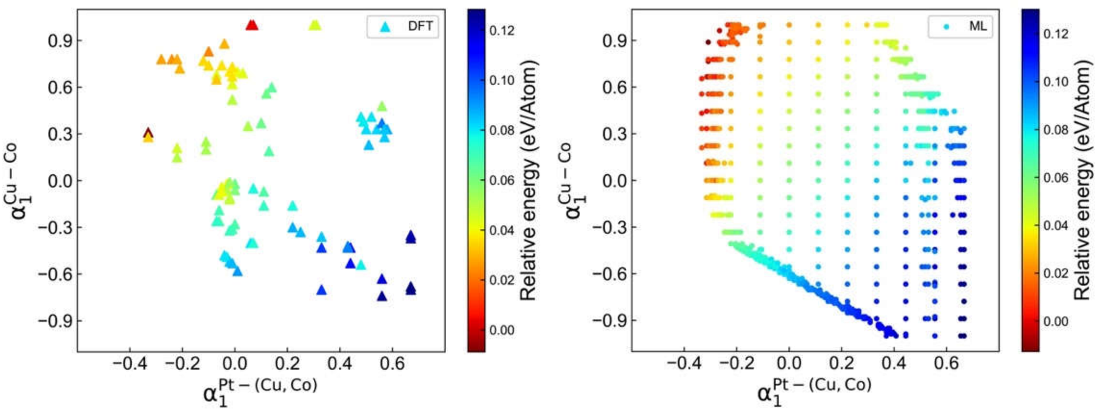

Supplementary Figure 9. DFT calculates (left) and ML predicted relative energies of Pt_2CoCu configurations as a function of Pt-Co/Cu SRO $\alpha^{Pt-(Co/Cu)}$ and Co-Cu SRO α^{Co-Cu}. The size of DFT calculated configuration and ML predicted are 120 and 3800 respectively.

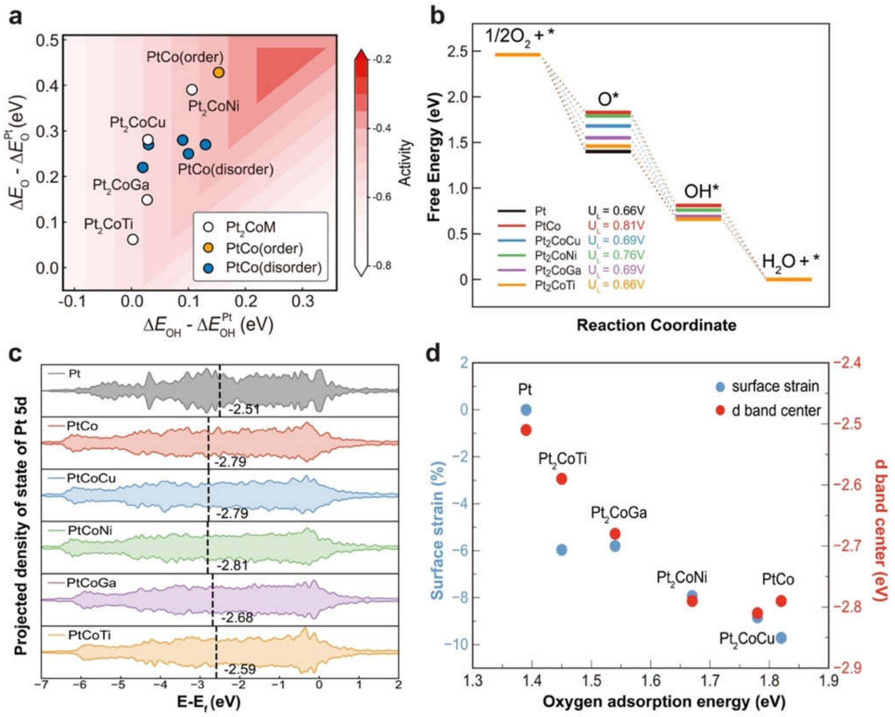

Supplementary Figure 10. ORR activity of Pt_2CoM based on DFT calculation. (a) Two-dimensional activity volcano plot for ORR activity as a function of OH and O adsorption energy relative to Pt. **(b)** Free energy diagram for oxygen reduction reaction for at U = 0 V, U_L depicts the highest potential where all reaction steps are exothermic. **(c)** The projected electronic density of states and d band center. **(d)** The linear relationship between oxygen adsorption energy with surface strain and d band center.

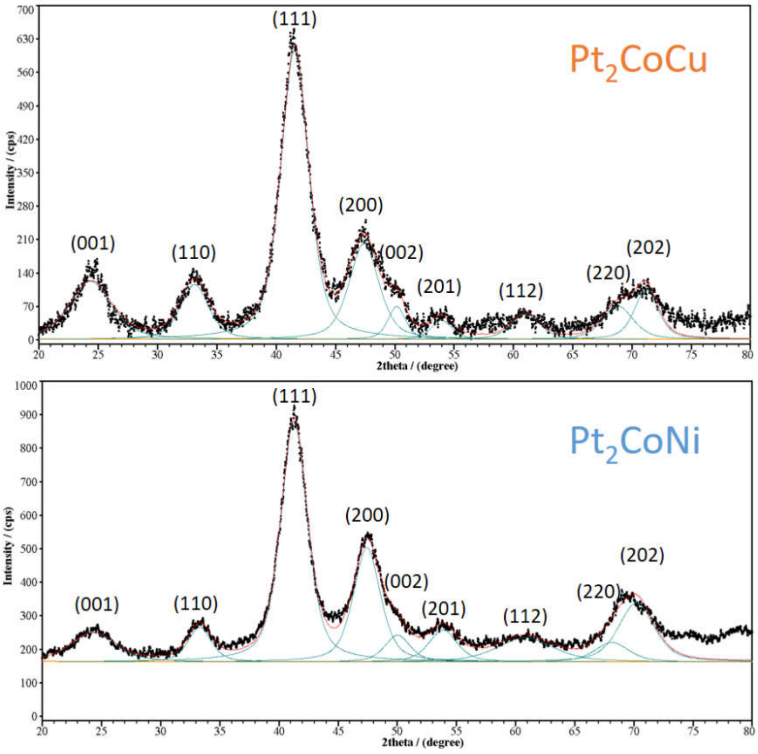

Supplementary Figure 11. Rietveld refinement of the raw XRD pattern of Pt_2CoCu (Ni).

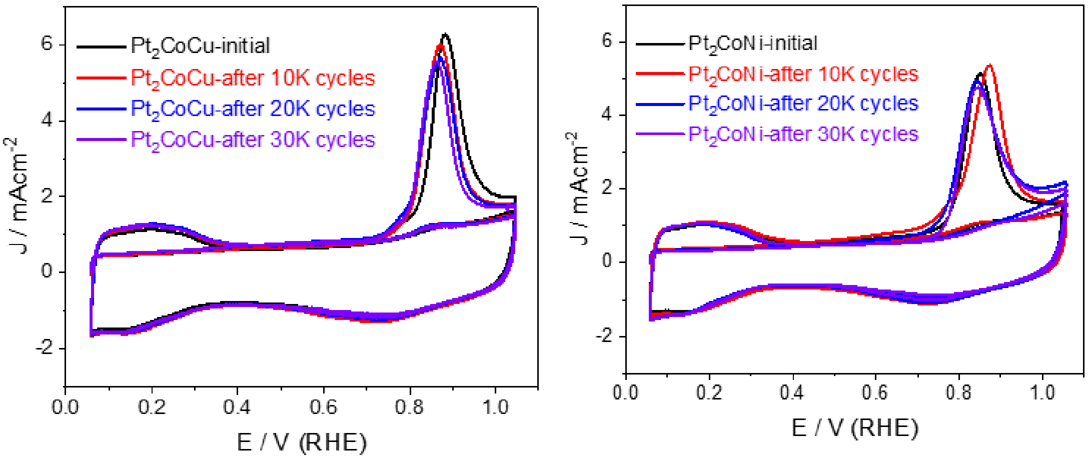

Supplementary Figure 12. CO-stripping curves of Pt_2CoCu (Ni) after each 10 K ADT.

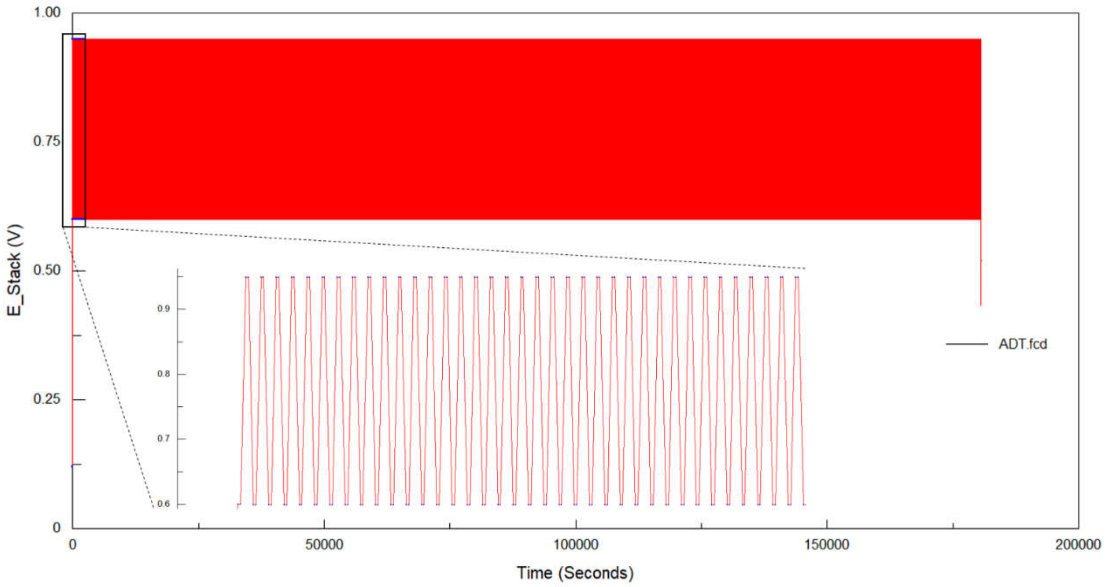

Supplementary Figure 13. 30K ADT protocol of a square wave potential cycling between 0.60 and 0.95 V (3-second hold at each voltage) suggested by US DOE.

Supplementary Table 1. Machine learning predicted ordering energy difference for Pt_2CoM combinations.

**IMC**	**Ordering Energy (eV/Atom)**
PtCo	0.0538
Pt_2CoCu	0.0652
Pt_2CoMn	0.0100
Pt_2CoGa	0.0542
Pt_2CoTi	0.0819
Pt_2CoNi	0.0647
Pt_2CoFe	0.0169

Supplementary Table 2. The size of DFT calculated configurations for the different Pt_2CoM.

IMC	Size (atoms)
PtCo	109
Pt_2CoCu	120
Pt_2CoNi	116
Pt_2CoMn	106
Pt_2CoFe	116
Pt_2CoGa	107
Pt_2CoTi	116

Supplementary Table 3. DFT calculated O and OH adsorption energies and limiting potential for 4 order Pt_2CoM combinations, order PtCo and disorder PtCo.

Alloy combination	ΔE_O (eV)	ΔE_{OH} (eV)	U_L (V)
Pt_2CoCu	1.67	0.94	0.69
Pt_2CoGa	1.54	0.94	0.69
Pt_2CoTi	1.45	0.91	0.66
Pt_2CoNi	1.78	1.02	0.76
PtCo (order)	1.82	1.06	0.81
PtCo (disorder)	1.66	1.04	0.75
PtCo (disorder)	1.64	1.01	0.69
PtCo (disorder)	1.61	0.93	0.68
PtCo (disorder)	1.66	0.94	0.76
PtCo (disorder)	1.67	1.00	0.79

Supplementary Table 4. Top view and side view of the DFT-optimized ORR intermediates adsorption configurations for order PtCo, Pt_2CoCu, Pt_2CoNi, Pt_2CoGa, Pt_2CoTi and disorder PtCo.

System	*	* OH	* O
PtCo (order)	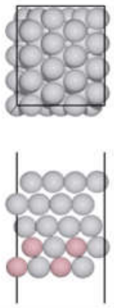	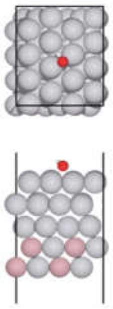	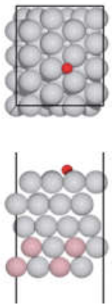
Pt_2CoCu	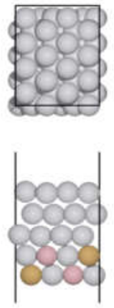	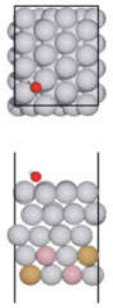	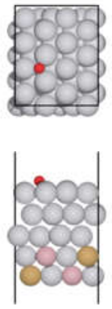
Pt_2CoNi	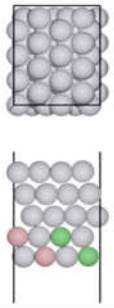	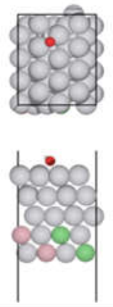	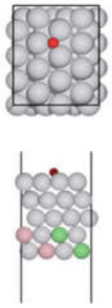
Pt_2CoGa	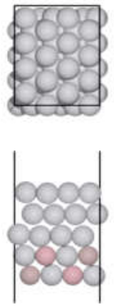	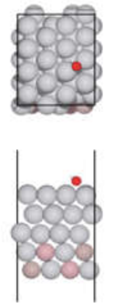	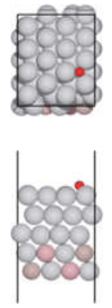

Pt_2CoTi	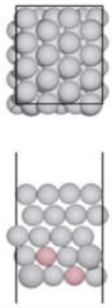	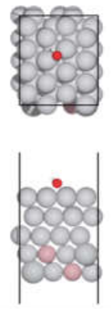	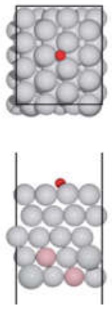
PtCo-1（disorder）	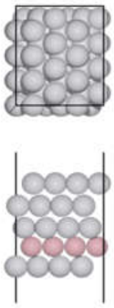	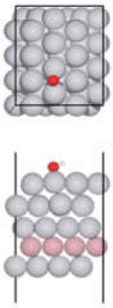	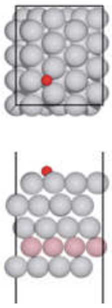
PtCo-2（disorder）	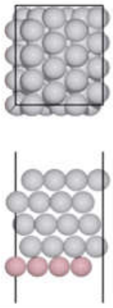	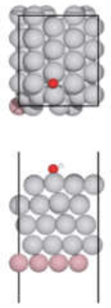	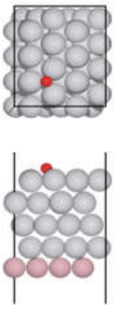
PtCo-3（disorder）	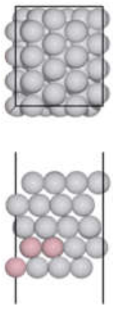	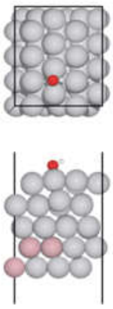	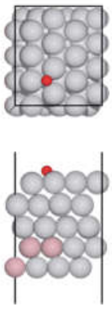
PtCo-4（disorder）	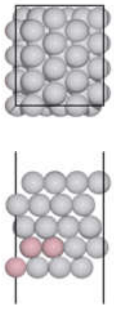	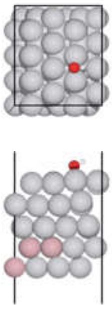	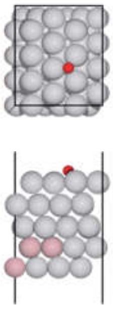
PtCo-5（disorder）	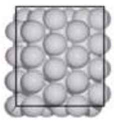	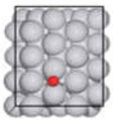	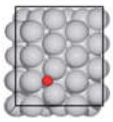

	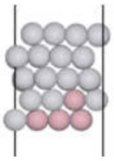	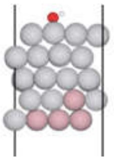	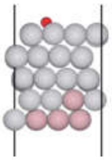

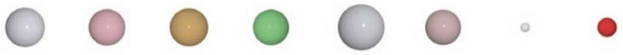
Pt
Co
Cu
Ni
Ti
Ga
H
O

Supplementary Table 5. Rietveld refinement of the XRD patterns and the corresponding parameter of Pt_2CoCu (Ni).

Pt_2CoCu

**hkl**	**2theta**	**FWHM**	**Height**
(001)	24.15	3.878	143
(110)	33.21	2.870	140
(111)	41.53	2.908	605
(200)	47.62	3.200	202
(002)	50.05	2.186	62
(201)	54.15	2.390	50
(112)	61.01	3.790	64
(220)	68.94	2.740	84
(202)	71.57	3.112	114

Pt_2CoNi

**hkl**	**2theta**	**FWHM**	**Height**
(001)	24.25	4.560	248
(110)	33.30	2.348	264
(111)	41.25	2.676	877
(200)	47.41	2.700	501
(002)	49.99	2.524	241
(201)	53.86	2.832	256
(112)	60.82	5.648	233
(220)	68.12	3.380	219
(202)	70.17	3.560	337

Supplementary Table 6. ECSA and SA of PtCo*, PtCo#, Pt_2CoCu, and Pt_2CoNi in the RDE test.

**Catalysts**	**ECSA**	**SA**
PtCo*	14.4 ± 2.2	3.41 ± 0.29
PtCo#	87.8 ± 4.7	1.67 ± 0.22
Pt_2CoCu	90.3 ± 3.5	3.69 ± 0.21
Pt_2CoNi	85.4 ± 4.1	3.4 ± 0.23

*large size particle with high ordering degree
#small size particle with low ordering degree
